# 

**Published:** 1886-07-20

**Authors:** 


### TABLE OF CONTENTS

ORIGINAL AND SELECTED ARTICLES.

Normal Labor Accompanied with Abnormal Phenomena in Mother and Child, by J. McF. Gaston, M D , Professor of the Principles and Practice of Su gery in the Southern Medical College, Atlanta, Georgia........ ....................245
A Few Grains and Some Chaff, from an Old Country Doctor ............ ................. .........249
A Case of Carcinoma of the Cervix Uteri, by Virgil O. Hardon, M.D., Lecturer upon Gynæcology in the So. Med. College, Atlanta, Ga...252
Malarial Fevers Treated by Quiniæ Sulph. Inunctions, by Dr J H. Young, Roswell, N. M..254
Drainage-Tube in the Chest for Twenty-one Months, by Oscar J. Coskery, M D..................256
At What Moment Does the Rational Soul Enter the Body, by John J. Gaynor, M.D., St. John, N. B...................................................... 258
Chionanthus Virginica, by J. A. Henning, M.D., Garnett, Kansas.............................................259
Inhalation in Pulmonary Tuberculosis; The Action of Quinine on the Fœtus; Kola ...........260

ABSTRACTS AND GLEANINGS.

The Turks......................................................261
Asthma.........................................................263
Experiments on a Decapitated Head..................264
New Method of Treating Whooping-Cough.....265
Gonorrhœa; A Classical Remedy for Hiccough..266
Chinese Medical Missions; Disguising the Taste of Quinine ..............................................267
Prescriptions for Sciatica; Earache ......... ...... 268
Rapid Cure of Suppuration of Middle Ear of Thirty five Years' Standing; White of an Egg Obstinate Diarrhœa..........................................269
Treatment of Acute Rheumatism ; Sparteine at Home; Cotton-Root Bark in Epilepsy........... 270
Some Forebodings of Incipient Insanity; Pilocarpin in Pneumonia; Brucine...................271
Membranous Croup; Chorea; Hot Flashes; The Incompatibility of Calomel and Bromide of Potassium................................ ..................272
The Digestibility of Various Kinds of Foods According to Vanderbeck; Cause of Fever; Salicylate of Soda in Sick Headache..................273
Antipyrin in Phthisis at Davos; Hystero-Catalepsy in a Male Patient; Alterative Medication........................................................274

SCIENTIFIC ITEMS.

The New Neubla in the Pleiades; Investigating Ghosts.................................................... .... 275
Fishing with Dynamite; What a Frenchman can do with a Hair; To Destroy Wood Worms ..........................................................276

PRACTICAL NOTES AND FORMULÆ.

For Cholera Morbus and Dysentery of Children ..............................................................277
Dysentery; For Impotence; Treatment of Acute Tonsillitis........... .......................... ....278

EDITORIALS AND MISCELLANEOUS.

A Liberal Offer; Editorial Notices............... .....279
Stone Mountain Medical Association...............281
Books and Pamphlets Received........................283
Special Notices...............................................284

